# “Self‐Peel‐Off” Transfer Produces Ultrathin Polyvinylidene‐Fluoride‐Based Flexible Nanodevices

**DOI:** 10.1002/advs.201600370

**Published:** 2017-02-23

**Authors:** Yanlong Tai, Gilles Lubineau

**Affiliations:** ^1^ Division of Physical Science and EngineeringKing Abdullah University of Science and Technology (KAUST)COHMAS LaboratoryThuwal23955‐6900Saudi Arabia

**Keywords:** polyvinylidene fluoride, “Self‐peel‐off” transfer (SPOT), ultrathin flexible nanodevices

## Abstract

Here, a new strategy, self‐peel‐off transfer, for the preparation of ultrathin flexible nanodevices made from polyvinylidene‐fluoride (PVDF) is reported. In this process, a functional pattern of nanoparticles is transferred via peeling from a temporary substrate to the final PVDF film. This peeling process takes advantage of the differences in the work of adhesion between the various layers (the PVDF layer, the nanoparticle‐pattern layer and the substrate layer) and of the high stresses generated by the differential thermal expansion of the layers. The work of adhesion is mainly guided by the basic physical/chemical properties of these layers and is highly sensitive to variations in temperature and moisture in the environment. The peeling technique is tested on a variety of PVDF‐based functional films using gold/palladium nanoparticles, carbon nanotubes, graphene oxide, and lithium iron phosphate. Several PVDF‐based flexible nanodevices are prepared, including a single‐sided wireless flexible humidity sensor in which PVDF is used as the substrate and a double‐sided flexible capacitor in which PVDF is used as the ferroelectric layer and the carrier layer. Results show that the nanodevices perform with high repeatability and stability. Self‐peel‐off transfer is a viable preparation strategy for the design and fabrication of flexible, ultrathin, and light‐weight nanodevices.

## 1 Introduction

Lithography is a well‐tested process that can be used to create flexible/printed electronics.^1^, ^2^, ^3^ Next‐generation flexible electronics are expected to perform under several constraints, including covering large areas at low cost, being lightweight/wearable, produced from environmentally friendly manufacturing processes, and easily integrated into other structures.^4^, ^5^ Up to now, apart from traditional photolithography, various lithographic methods, such as inkjet printing,^6^ atomic layer lithography,^7^ electrohydrodynamic lithography,^8^ soft lithography,^9^ constructive lithography,^10^ and adhesive lithography,^11^ have been explored in pursuit of a cheaper, greener, and simpler fabrication alternative. Among these methods, adhesive lithography has been shown to be very efficient and scalable; moreover, it is less sensitive to the formula of the ink and to the substrate surface treatment than are other lithographic methods.^11^, ^12^ Polydimethylsiloxane (PDMS) and poly(urethaneacrylate) are the most common polymers used in adhesive lithography to produce stretchable devices.^12^, ^13^ Until now, however, adhesive lithography has not been used to fabricate flexible substrates that do not stretch, even though such substrates have broader potential applications in flexible electronics. Nonstretchable but flexible electronics can find important applications in wearable electronics: flexibility is required to match the shape‐changing fabrics, but stretchability is often not needed and could even damage the device. One potential substrate that does not stretch that can be used in flexible electronics is polyvinylidene‐fluoride (PVDF).

Functional materials are sensitive to variations in environmental humidity, temperature, light, and pH, along with changes in the physical or chemical performance of the material itself.^14^, ^15^ Typically, the polymer chain of PVDF, (the repeated unit of –(CH_2_CF_2_)*_n_*–)) curls in the presence of water molecules, greatly reducing its adhesive capability^16^, ^17^ and rendering it unattractive as an adhesive agent. The curling results from the quite different polarities between the chemical groups of R‐F and R‐H (R: polymer chain), as well as the repulsive force between the fluorine atoms, which produces the change in the chain configuration.^18^ For instance, during the preparation of lithium ion batteries in which PVDF is used as both a separator and a bonder, humidity control is essential to maintaining PVDF's adhesion.^19^, ^20^ PVDF experiences high deformations when subjected to changes in temperature due to its high coefficient of thermal expansion (CTE, α = 127 × 10^−6^ K^−1^), which easily generates internal stress in laminated films containing a PVDF layer.^21^ Carbon materials (e.g., single‐walled carbon nanotube (SWCNT), graphene oxide (GO)) have negative CTEs (α = −1.5 × 10^−6^ and −67 × 10^−6^ K^−1^, respectively), which means that these materials contract whereas PVDF expands when the environmental temperature increases.^22^ As far as moisture absorption is concerned, both PVDF and carbon material expand when they take up water.^23^ We confirmed these phenomena in a SWCNT/PVDF bilayer film, which exhibited dramatic actuation behaviors when exposed to light because of the opposite deformation of each material as a response to variation in environmental humidity or temperature.^24^ Inspired by the possibilities of adhesive lithography and by the potential of PVDF to exhibit large dimensional changes in response to changes in temperature, we fabricated flexible ultrathin nanodevices using a new technique, “self‐peel‐off” transfer (SPOT).

**Figure 1:**
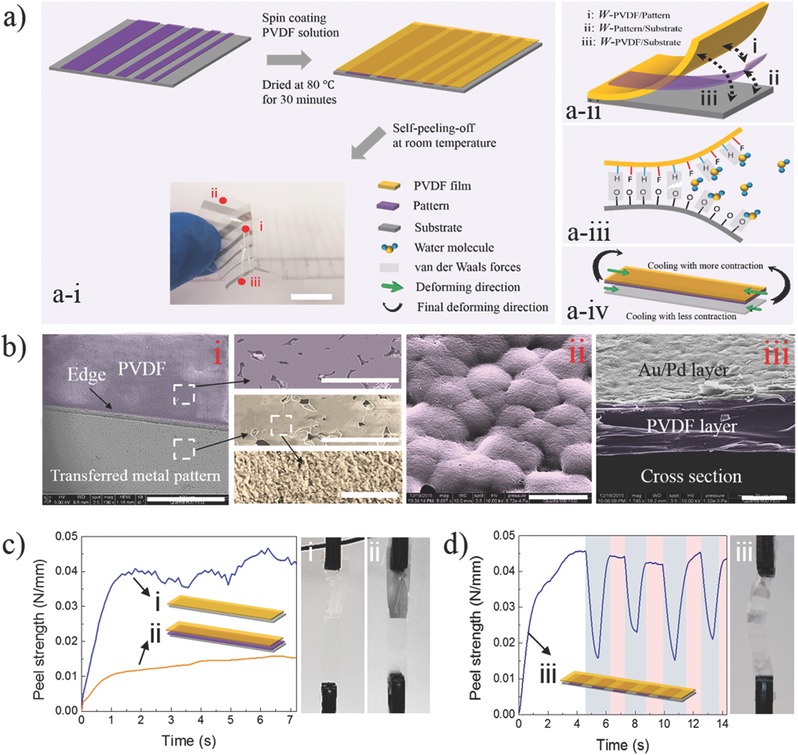
a) Schematic illustration of the fabrication of an ultrathin flexible PVDF film using SPOT (a‐i) and its mechanism (a‐ii, a‐iii, a‐iv, respectively); the scale bar is 1 cm; b) SEM images of the transferred flexible PVDF film at different points and magnifications (i: the transferred metallic film; ii: PVDF film; iii: cross‐section of the Au‐Pd/PVDF film); the scale bars are 500, 20, 20, and 1 µm for image‐i, 5 µm for image‐ii, 20 µm for image‐iii; c,d) peel‐off test applied to typical flexible films with different structures along with peel curves and digital images (i: PVDF‐polyethylene terephthalate (PET); ii: PVDF‐Pattern(continuous)‐PET; iii: PVDF‐Pattern(discontinuous)‐PET). The sample size for the peel‐off test was 6 cm × 8 mm. The thickness of the PVDF layer was 25 ± 3 µm. The thickness of the PET layer was 50 µm. The default peel‐off speed was 1 cm min^−1^.

In this paper, we first show that PVDF can be used as an adhesive material to transfer functional patterns for the fabrication of ultrathin flexible nanodevices, as shown in **Figures** **1**a,b. To ensure a successful peel‐off process, we then tune the work of adhesion between the different layers (*W‐*_PVDF/substrate_, *W‐*_PVDF/pattern_, and *W‐*_pattern/substrate_) via variation in environmental humidity. Delamination is triggered by the interlaminar stresses generated by the differential dilatation between the layers, revealing the theoretical basis of the self‐peel‐off mechanism.

Second, we use SPOT to fabricate flexible films from gold‐palladium (Au‐Pd)/PVDF. Results from our morphological analyses, including surface profilometry, resistance measurements (before and after the transfer process) and dynamic mechanical tests, demonstrate the success of this technique, as well as the adjustable capability of the work of adhesion in the fabrication of SWCNT/PVDF film. We also test the technique on other particles and substrates.

Third, we explore a series of practical applications. We report on our development of a wireless humidity sensor, regarded as a single‐sided flexible device, with an Au‐Pd‐based pattern as the antenna and a SWCNT film as the humidity‐sensitive layer, and an ultrathin capacitor, regarded as a double‐sided flexible device, with the Au‐Pd and SWCNT layers serving as electrodes.

## 2 Results and Discussion

### 2.1 Mechanism Consideration

SPOT is a promising method for the fabrication of flexible electronics. Three conditions are required in the SPOT process: first, different works of adhesion (W‐_polymer/pattern_ > W‐_pattern/substrate_) are necessary to ensure that the pattern on the substrate can be transferred to the polymer film during the peel‐off phase; second, the polymer film should be easily peeled off from the substrate, which requires that W‐_polymer/substrate_ is low; third, a driving interlaminar force should be generated from the flexible film to trigger the self‐peel‐off mechanism.

In the first condition, the work of adhesion between nanomaterials and substrates (*W*‐_pattern/substrate_) is very weak because it is provided by van der Waals' forces.^25^ Usually, various strategies are used to improve the work of adhesion, such as the addition of an adhesive agent in the nanomaterial formula,^26^ sintering at high temperature,^27^ presurface treatment of the substrate,^28^ or sputter deposition with high energy.^29^ Even with such strategies, *W*‐_pattern/substrate_ remains small with respect to *W*‐_polymer/pattern_ if PVDF is used as the polymer. This is because the PVDF solution is dropped on the nanoparticles. The adhesive force can be increased if the PVDF polymer chains are allowed to coat the surface of the nanoparticles in solution and then to grasp them via hydrogen bonding or physical absorption after a curing/drying process. Moreover, the adhesive force can even be further increased after plasma treatment on the pattern.^30^

In condition 2, the adhesion between the polymer layer and the substrate (polymer, wafer, glass, etc.) is very strong after the curing/drying process due to the adsorption/entanglement of the polymer chains onto the substrates, as confirmed via a peel‐off test on laminated composites.^31^, ^32^ How this adhesive force is controlled is crucial to SPOT. We need to reduce the work of adhesion between the polymer layer and the substrate without reducing *W*‐_polymer/pattern_; if we were to reduce *W*‐_polymer/pattern_, then we would not be able to meet condition 1. This is the main barrier in the fabrication of flexible devices via adhesive lithography.

PVDF provides a perfect solution to this problem compared with other polymer materials. First, PVDF, an effective bonder, has well‐separated polymer chains, which can sufficiently immerse into and coat the surface of a substrate via physical absorption or hydrogen bonding.^32^ Uniquely, its polymer chains curl after exposure to high humidity, decreasing its adhesive capability.^33^ In that process, water molecules weaken the interaction between PVDF and the substrate, as shown in Figure 1a‐iii. After drying at 80 °C (relative humidity, RH = 18.6%) and cooling in the ambient environment (24.3 °C, RH = 43.1%), *W*‐_PVDF/substrate_ should decrease due to the variation in humidity (Δ*C*), allowing the PVDF film to be easily peeled off. Yet, the interface between PVDF and the pattern remains tough because the particles are largely embedded in the PVDF layer, resulting in *W*‐_PVDF/pattern_ > *W*‐_PVDF/substrate_. We confirmed the capability of the peel test with samples of PVDF/polyethylene terephthalate (PET), PVDF/Au‐Pd(continuous)//PET, and PVDF/Au‐Pd(discontinuous)/PET, as shown in Figures 1c,d. The peel‐off strength was below 0.045 N mm^−1^, which is much lower than an earlier reported value (0.4–0.6 N mm^−1^).^33^ Thus, we infer that PVDF is not only a great adhesive material, but it is also easily peeled off during the film fabrication process when environmental conditions are favorable.

In condition 3, the self‐peel‐off driving force is generated via the differences in thermal expansion between the adjacent layers. PVDF has a high CTE (α = 127 × 10^−6^ K^−1^) compared with the value for glass (9 × 10^−6^ K^−1^) and for typical PET (40–50 × 10^−6^ K^−1^). The PVDF solution first dries at high temperature. During cooling at room temperature (Δ*T*), the substrate contracts but the PVDF layer contracts more, as seen in Figure 1a‐iv. This is the main mechanism leading to the development of high interlaminar stresses. Of course, during the cooling at room temperature, the laminate experiences both thermal and hydric expansion as both the temperature and the relative humidity change. We can safely attribute most of the observed expansion to the change in temperature as the coefficients of hygroscopic expansion of these materials are rather low when compared to their CTE values, as seen in Table S1 (Supporting Information).

**Figure 2:**
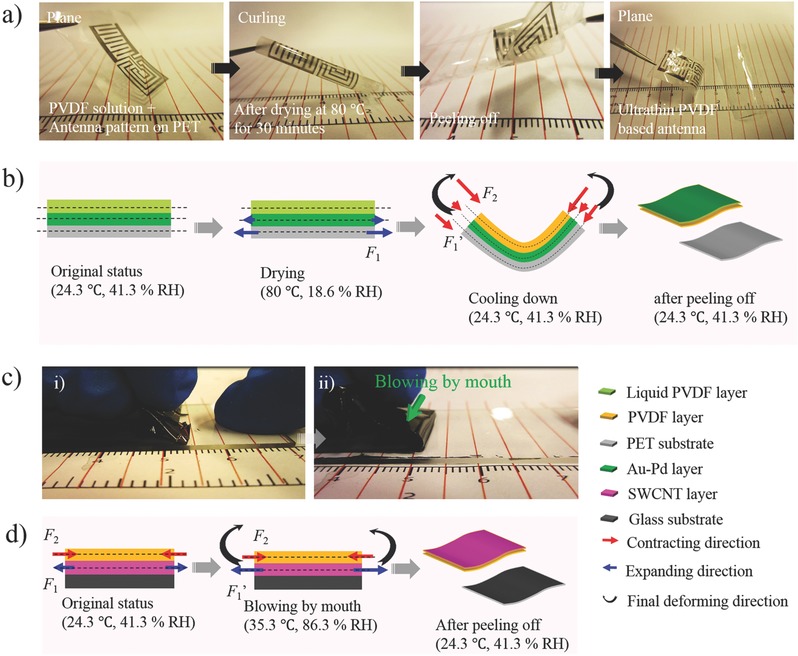
Mechanism of SPOT technique. a) Digital images of a typical fabrication process of a complex flexible Au‐Pd/PVDF antenna; b) tracking of the corresponding variation in the internal stress during the whole fabrication process; c) digital images of a typical preparation of a large‐area flexible SWCNT/PVDF film, i) before and ii) after blowing by mouth; d) tracking of the corresponding variation in the internal stress during the whole fabrication process. A glass substrate was used to avoid influence from the substrate.

The mechanism is further illustrated via the fabrication of the complex Au‐Pd/PVDF antenna shown in **Figure** **2**a,b and Video S1 (Supporting Information). As can be seen, the dried freestanding film curled, indicating that, during cooling, high residual stresses appear between the PVDF and PET layers. These residual stresses are relaxed after the peel‐off process as the freestanding film recovers a flat geometry after peel‐off. When high enough, these residual stresses produce interlaminar delamination and self‐peel‐off occurs.^34^, ^35^ We note that the curvature of the laminate after cooling is directly related to the intensity of these residual stresses. The relation between the radius of curvature and the physical properties of a bilayer laminate is described in Equation (1), 36

(1) $$\frac{1}{R_{T}}\;-\;\frac{1}{R_{T_{0}}}\;=\;k\frac{T-T_{0}}{s}\left(k\;=\;\frac{6(\alpha_{i}\;-\;\alpha_{j}){\left(1\;+\;m\right)}^{2}}{3{\left(1\;+\;m\right)}^{2}\;+\;(1\;+\;m\cdot n)\left(m^{2}\;+\;\frac{1}{m\cdot n}\right)}\right)\;$$

where i and j are the materials of each layer; α_i_ and α_j_ are the CTEs of materials i and j, respectively; Δ*T* is the change in environmental temperature; *R_T_* is the radius of curvature at the final temperature, *T*, and *R_T_*_0_ is the radius of curvature at the starting temperature, *T*_0_. *m* and *n* are defined as *m* = *t*_i_/*t*_j_ (the ratio of the thicknesses of the two layers) and *n* = *E*_i_/*E*_j_ (the ratio of the elastic moduli of materials i and j). *s* is the total thickness of the film (*s* = *t*_i_ + *t*_j_).

Further analysis on this mechanism is provided in the Supporting Information (Figure S1) and Supporting Information text.

To complete this analysis, we prepared flexible PVDF films with different materials, including SWCNT, GO, and lithium iron phosphate. We present details of the SWCNT/PVDF film in Figure 2c,d, and Video S2 (Supporting Information). Specifically, the SWCNT/PVDF film cannot be peeled‐off directly after cooling down, similar to the Au‐Pd/PVDF film, though it has a negative CTE (−1.5 × 10^−6^ K^−1^). This may be because of the stronger adhesive force between the SWCNT layer and the glass substrate. However, after we blew on the film, we could peel it off easily because the humidity in the environment increased from 41.3% RH to 84.7% RH from our breath, leading to further expansion of the SWCNT layer. The final internal stress became strong enough to overcome the adhesion. We previously identified this as the main mechanism of an ultrasensitive actuator.^24^ In short, the peel‐off process comprises both chemical and physical actions influenced by temperature and humidity. We can thus tune interfacial stresses to improve the peel‐off process.

For comparison, we tested other polymers, including PDMS and standard adhesive tape, as shown in Figure S2 (Supporting Information). Results indicated that these other polymers performed poorly. Generally, we attribute the success of the flexible film made from PVDF via SPOT to the physical and chemical properties of PVDF.

### 2.2 Characterization of an Au‐Pt/PVDF Flexible Film Prepared via SPOT

**Figure 3:**
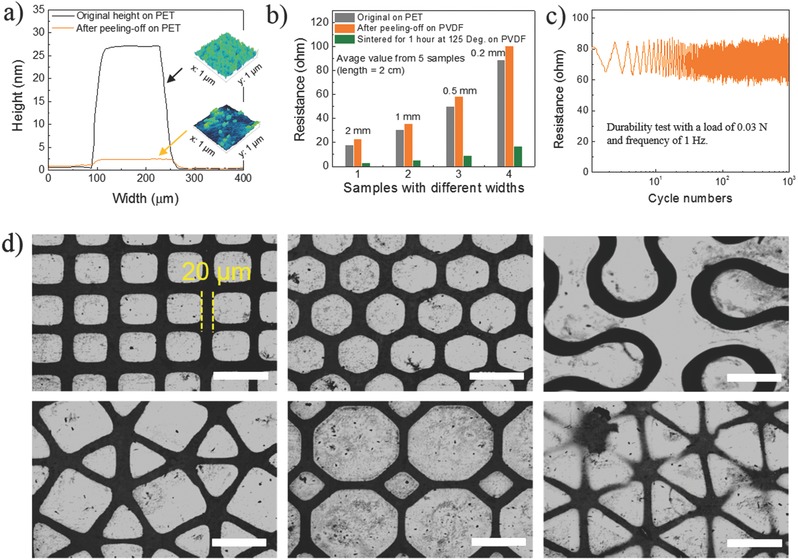
Performance of flexible Au‐Pd/PVDF films fabricated via SPOT. Variations in a) the surface‐profile (insets are AFM images of Au‐Pd particles on PET substrate before and after the peel‐off process) and b) sheet resistance of Au‐Pd patterned on PET or PVDF substrates before and after the transfer process; c) dynamic mechanical properties of flexible Au‐Pd/PVDF films with a sample size of 4 cm × 8 mm × 30 µm; d) various Au‐Pd/PVDF film patterns prepared via SPOT. All the scale bars are 100 µm.

To further confirm the efficiency of SPOT, we compared the surface profiles of lines of the Au‐Pd deposited on PET before and after peeling. The results are presented in **Figure** **3**a and the inset atomic force microscopy (AFM) images. These results show that the deposited lines of Au‐Pd almost totally transferred from the original PET substrate to the PVDF film. Indeed, the height of Au‐Pd particles on the PET substrate changed from 27.2 nm to around 3 nm. More details can be seen in the SEM images in Figure S3 (Supporting Information). The residual Au‐Pd film left on the PET substrate comes not only from the roughness of the PET film itself but also from the microholes in the PVDF film, which may reduce the interactions between PVDF and the Au‐Pd particles. This is confirmed in the microstructure images in Figure 1b in which holes (500 nm – 1 µm in size) can be observed on both sides of the PVDF film. These holes can be reduced or even avoided by controlling the humidity during the drying process.^37^, ^38^ The defects caused by these holes are also captured in the conductive performance of the lines of Au‐Pd. As shown in Figure 3b, the resistance of the transferred conductive Au‐Pd lines on the PVDF substrate increased by about 7.1%, 5.2%, 4.0%, 3.1% (Δ*R*/*R*_0_, Δ*R* = *R*_PVDF_−*R*_PET_) more than that of the resistance of the original conductive Au‐Pd lines on the PET substrate, with line widths of 200, 500, 1000, 2000 µm, respectively. This increase in resistance results from both the nearly complete transfer of the nanoparticles (Figure 3a) and the change in the morphology of the conductive layer due to the surface defects on the PVDF substrate (Figure 1b). The change in resistance varies slightly depending on the line width because the surface defects on the PVDF substrate (which are holes with the same diameter) will generate more significant effects on thinner lines. However, the conductive performance of the lines of Au‐Pd vastly improved (with a resistance about 6–7 times lower than the original resistance on the PVDF substrate) after sintering at 150 °C for 30 min (Figure 3b).^39^ This improved performance results from the rearrangement of the Au‐Pd nanoparticles into larger particles during sintering, which reduces electron scattering at the interfaces between the particles, as seen in Figure S4 (Supporting Information).

Due to the low surface energy of PVDF, the interfacial stability between PVDF and lines of Au‐Pd must be considered, as delamination could arise after long‐term use. To determine the durability, we tested a 4 cm × 0.8 cm flexible Au‐Pd/PVDF film. It maintained a clear signal after 1000 cycles with negligible changes to the resistance amplitude, indicating excellent mechanical durability with no noticeable drift, as shown in Figure 3c. The mechanical durability mainly results from the good coating of the Au‐Pd nanoparticles by PVDF. We used a low‐molecular‐weight PVDF (average *M*_n_: 71 000) to ensure good coverage.

In addition, various complex patterns with the thinnest line width of 20 µm were also prepared via SPOT as shown in Figure 3d. As shown, the patterns are high quality, further indicating the versatility and repeatability of this technique. Moreover, the resolution of SPOT can be further improved, low to nanoscale in theory.

### 2.3 A Single‐Sided Flexible Device

To verify the practical application of the SPOT technique, we prepared a complex Au‐Pd antenna, which can be regarded as a single‐sided flexible device, aiming to make full use of the excellent dielectric property of PVDF as the substrate. All geometrical parameters of a typical antenna sample are presented in the Experimental Section and the processing is captured on Video S1 (Supporting Information).^40^, ^41^

**Figure 4:**
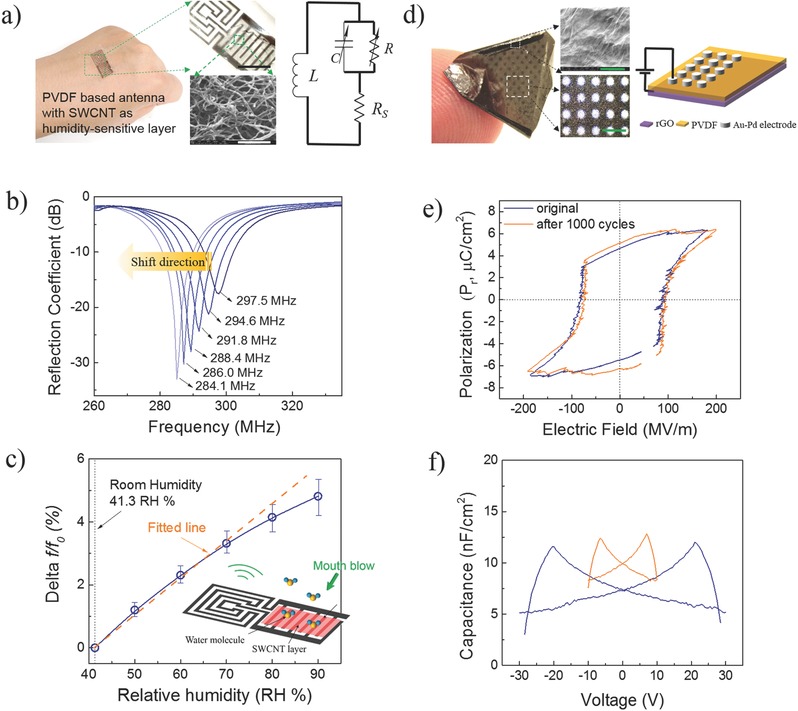
a) Digital images and schematic of an equivalent electric circuit of a typical PVDF‐based wireless humidity sensor with a SWCNT film as the humidity detection layer, attached to a human hand; scale bars are 1 cm and 1 µm, respectively; b) frequency response in the reflection coefficient of the sensor to various humid environments (41.3% RH, 50% RH, 60% RH, 70% RH, 80% RH, 90% RH, from right to left); c) the relevant variation in the resonant frequency of the sensor to various humidity environments (inset: the sensor's working mechanism); we note that the various humidity environments were created by blowing by mouth. Fabrication and performance of a flexible ultrathin PVDF capacitor via SPOT; d) photograph of a typical flexible capacitor with magnified images of the rGO and Au‐Pd electrodes; scale bars are 1 µm and 1 mm, respectively, and a schematic of the device's architecture; e) polarization versus voltage (*P*−*V*) curves before and after 1000 dynamic cycles; f) capacitance versus voltage (*C*–*V*) with different applied voltages.

After an ultrathin SWCNT is integrated into the staggered electrode area, this device forms a complete LCR (Inductor–Capacitor–Resistor) oscillator, which can be used to monitor changes in environmental humidity due to the strong humidity‐sensitive properties of the SWCNT layer.^27^ The sensor can be seen in **Figure** **4**a with the relevant equivalent electric circuit. More fabrication details are provided in the Supporting Information.

The results, summarized in Figure 4b, demonstrate that our single‐sided device has a resonant frequency of 297.5 MHz under ambient humidity of 41.3% RH. The resonance shifts to a lower frequency when the humidity increases. Specifically, when the humidity changes to 50% RH, 60% RH, 70% RH, 80% RH, 90% RH, the resonant frequency shifts to 294.6, 291.8, 288.4, 286.0, 284.1 MHz, respectively. Indeed, higher humidity increases the capacitive part (*C*) of the SWCNT layer and finally results in a decrease in *f*_sensor_.

These results are further summarized in Figure 4c in which the sensitivity of LCR circuit systems is evaluated by the variation in resonant frequency (Delta *f/f*_0_) in relation to the relative humidity (RH%). Results show that the sensitivity of this device is up to 0.125 RH^−1^, which is high enough to be used in wireless wearable electronics.

### 2.4 A Double‐Sided Flexible Device

To further verify the practical application of our SPOT technique, we prepared an ultrathin flexible capacitor (total thickness: 3 ± 0.5 µm) with an Au‐Pd circular pattern as one electrode, reduced graphene oxide (rGO) as the other electrode, and PVDF as the ferroelectric layer and carrier layer. More details are provided in the Experimental Section of the Supporting Information. A typical double‐sided capacitor with its architecture is presented in Figure 4d. The sandwich structure can be regarded as a double‐sided flexible device that takes full advantage of the ferroelectricity of PVDF.^42^ The polarization of the PVDF film mainly results from the enriched chemical groups (e.g, carbonyl and hydroxyl groups) on the rGO film. These chemical groups interact with the fluorine groups in the PVDF chain.^43^ The development of the β‐phase is confirmed via X‐ray powder diffraction (XRD) in Figure S5 (Supporting Information).^44^

The hysteresis loops of a typical capacitor are shown in Figure 4e, where the displaced charge density is presented as a function of the electric field with a fixed frequency of 100 Hz. A full hysteresis loop with a constant remnant polarization of 6.49 µC cm^−2^ is obtained at an electric field of about 190 MV m^−1^. After 1000 dynamic‐mechanical cycles, the sample shows a similar profile (the dynamic‐mechanical test is described in Figure 3c). This result further confirms the good adhesion among the three layers resulting from the SPOT method.

In addition, we also characterized the capacitance versus voltage (*C*–*V*) responses of the double‐sided flexible film as shown in Figure 4f. Hysteresis with a butterfly shape can be observed. This phenomenon results from the irreversible ferroelectric polarization of the PVDF layer from dipole rotation in the presence of a bias voltage.^45^ The double‐sided Au‐Pd/PVDF/rGO capacitor prepared by SPOT clearly performs well, indicating promising applications in information‐storage devices.

## 3 Conclusion

In conclusion, we introduced a new strategy for the preparation of unstretchable, ultrathin PVDF‐based nanodevices via the SPOT technique. We analyzed the mechanism of this technique using the different works of adhesion between the different layers, directly resulting from the basic physical/chemical properties of PVDF and nanomaterials. The high performance of the resulting functional nanomaterial/PVDF films provided further confirmation of the utility of the technique. In addition, we prepared a single‐sided wireless flexible humidity sensor in which PVDF was used as the substrate and a double‐sided flexible capacitor in which PVDF was used as the ferroelectric layer and the carrier layer. The high performance of these devices indicates that this strategy is a viable new path for the design and fabrication of flexible, ultrathin, and light‐weight PVDF devices (with theoretical thicknesses to the nanoscale). Further investigations are necessary to demonstrate more practical applications, such as a nanogenerator based on the piezoelectric property of PVDF, an actuator exploiting the high CTE of PVDF, as well as a freestanding lithium‐ion battery based on the microcellular structure of PVDF.

## 4 Experimental Section

*Materials*: SWCNTs (with an outer diameter of 1–2 nm, a length of 5–30 µm, and over 95 wt% purity and 2.56 wt% COOH groups) were purchased from CheapTubes, Inc. The ink (1 mg mL^−1^) was prepared in the laboratory according to the previous methods.^24^ Graphite oxide ink (0.1 mg mL^−1^) was synthesized and prepared in the laboratory from purified natural graphite (SP‐1, Bay Carbon) by the Hummers method. Lithium iron phosphate (LiFePO_4_) paste (diameter of LiFePO_4_ powder = 300 nm, solid content = 45 wt%) was purchased from Tatung Co. PVDF (average *M*_n_ = 71 000) and N, N‐dimethylformamide (DMF) were purchased from Sigma‐Aldrich. PET films (with a thickness of 50 µm) were purchased from Teonex Inc. Deionized water was used in all experimental processes. The main raw materials are pictured in Figure S6 (Supporting Information).

*Fabrication of a Flexible PVDF Device*: To make the PVDF solution, we combined PVDF powder (30 g) and DMF (70 g) in a glass bottle. Then, the mixture was homogenized using a Brason 8510 bath sonicator (Thomas Scientific) for 1 h, followed by magnetic stirring at a high speed on a hotplate (80 °C) until a transparent and homogeneous yellow solution was obtained (about 8 h). The final PVDF solution was 30 wt%.

To make the flexible PVDF film, we deposited Au‐Pd on an ethanol‐cleaned PET film using a sputter instrument with a current of 20 mA and a duration of 180 s. After drying at 80 °C for 20 min in a hot oven to remove the absorbed moisture (high temperatures are not possible due to the low melting point of the Au‐Pd nanoparticles), a PVDF layer was fabricated on the surface of the Au‐Pd layer by the spin‐coating technique. Then, the layered film was dried for 30 min under the same conditions. Next, the fabricated sandwich structure (PET/Au‐Pd/PVDF) was easily peeled off or layered spontaneously. The Au‐Pd layer transferred from the PET substrate to the PVDF film, regardless of the thickness of the PVDF layer. A typical ultrathin Au‐Pd/PVDF film is shown in Figure 1a.

We also fabricated flexible PVDF films layered with other nanomaterials (SWCNT, GO, and LiFePO_4_) using the same procedures, as shown in Figures S7 and S8 (Supporting Information). Flexible PVDF films with various patterns were designed using computer‐aided‐design software and assisted via a wafer template.

To prepare the single‐sided flexible PVDF sensor, an antenna pattern was created using the technique described above. More details are provided in Figures S9 and S2a, and Video S2 (Suppporting Information). To improve its conductivity, this PVDF antenna was sintered at 150 °C for 1 h and the final total resistance was reduced to 182 ohms. Second, the SWCNT layer was deposited through dropping the SWCNT ink (1 mg mL^−1^) on a plasma‐treated, staggered electrode using a Thermo Scientific Finnpipette (0.2–2 µL) with a controlled concentration of 2 µL cm^−2^ and then baked in a hot oven at 80 °C for 30 min. The sensor is shown in Figure 2a.

To make the double‐sided flexible capacitor, we prepared a SWCNT/PVDF flexible film using the technique described above. We controlled the thickness of the PVDF layer to 3 ± 0.5 µm via a spin speed of 2000 rpm for 30 s. Next, Au‐Pd circlular array electrodes (diameter = 300 µm) were fabricated via sputter deposition (current = 20 mA for 180 s) using a template. The capacitor is shown in Figure 4d and Figure S8 (Supporting Information).

*Characterization and Measurements*: The flexible PVDF films fabricated via SPOT were examined by a scanning electron microscope (Quanta 600, FEI Company) for surface morphology analysis, a surface profilometer (Veeco Dektak 150) operating at a scanning speed of 0.167 µm s^−1^ and AFM (Veeco, Dimension 3100) for the assessment of transferring efficiency, a 4‐point probe system (Pro4‐440N, Lucas Labs) for sheet resistance measurement, a PC‐controlled universal test machine (Instron 5944 with a 5‐N load cell) with a PC‐recordable multimeter (Agilent 34401A) for the peel‐off test and cyclic mechanical tests.

The prepared wireless flexible humidity sensor, which can be considered as an LCR circuit, was characterized by a PNA network analyzer (Agilent N5225A, 10 MHz–50 GHz) with a coaxial cable and a sub‐miniature version A connector for the analysis of the resonant frequency (*f*_sensor_) and the relevant reflection coefficient. Due to the strong humidity sensitivity of the carbon nanotubes, the capacitance (*C*) in the LCR circuit changes, resulting in an *f*_sensor_ shift according to Equation (2)

(2) $$f_{\mathrm{sensor}}=\frac{1}{2\Pi \sqrt{LC}}$$

Blowing through the mouth was used as the humidity source, and the relative humidity was thoroughly mapped using a humidity meter (TM325, Dickson). The default was the ambient environment with a RH of 41.3% and temperature of 24.3 °C.

The flexible SWCNT/PVDF/Au‐Pd capacitor was characterized using a Premier Precision II ferroelectric tester (Radiant Technologies Inc.) to establish the polarization (*P*) – voltage (*V*) curves and a semiconductor characterization system (4200‐SCS, Keithley company) with a Cascade Microtech (Summit‐11600 AP) microprobe station to determine the capacitance (*C*) – voltage (*V*) curves.

## Supporting information

As a service to our authors and readers, this journal provides supporting information supplied by the authors. Such materials are peer reviewed and may be re‐organized for online delivery, but are not copy‐edited or typeset. Technical support issues arising from supporting information (other than missing files) should be addressed to the authors.

Supplementary

Supplementary

Supplementary
