# Supplementary material for: “Self‐Peel‐Off” Transfer Produces Ultrathin Polyvinylidene‐Fluoride‐Based Flexible Nanodevices
Source: Adv Sci (Weinh). 2017 Feb 23;4(4):1600370. doi: 10.1002/advs.201600370 (PMC5396151; doi:10.1002/advs.201600370)
Supplement: Supplementary file 1 — Supplementary [file ADVS-4-na-s001.pdf]

Copyright WILEY-VCH Verlag GmbH & Co. KGaA, 69469 Weinheim, Germany, 2016.

Supporting Information

for *Adv. Sci.,* DOI: 10.1002/advs.201600370

"Self-Peel-Off" Transfer Produces Ultrathin Polyvinylidene-Fluoride-Based Flexible Nanodevices

Yanlong Tai and Gilles Lubineau**

((Supporting Information can be included here using this template))

Copyright WILEY-VCH Verlag GmbH & Co. KGaA, 69469 Weinheim, Germany, 2013.

Supporting Information

“Self-Peel-Off” Transfer Produces Ultrathin Polyvinylidene-Fluoride-based Flexible Nanodevices

*Yanlong Tai, Gilles Lubineau**

This PDF file includes:

1. Videos of experimental work

   Video-S1: The preparation of a typical flexible Au-Pd/PVDF antenna via SPOT.

   Video-S2: The preparation of a typical flexible SWCNT/PVDF film via SPOT (with/without blowing by mouth).

2. Analysis of the mechanism of SPOT in the Supplementary Text, Scheme S1, Figure S1, and Table S1.
3. Further characterization of experimental samples and method for Figures S2 to S9.
4. References.

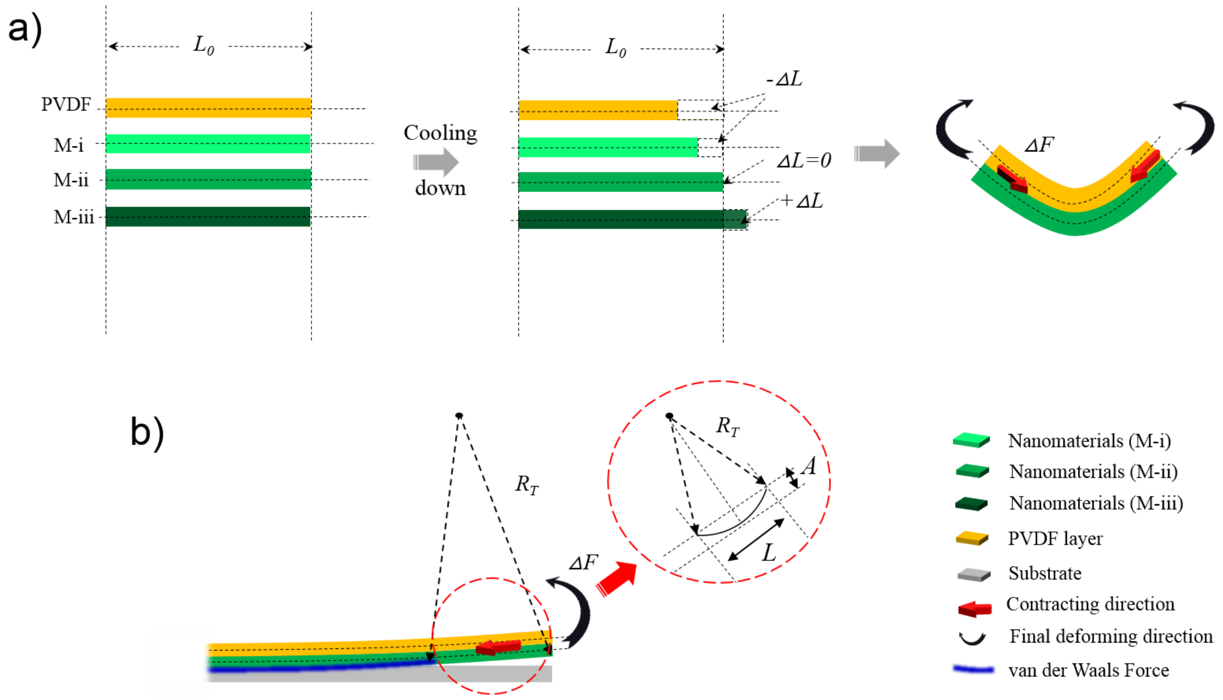

Scheme S1. *Fabrication of PVDF-based bilayer films using SPOT. a) Explanation of the stress response of PVDF-based films; b) The self-peel-off behavior of PVDF-based films attached on a PET substrate via van der Waals forces. (R_T: the radius of curvature at temperature T; A: the deflection of PVDF based films; ΔF: the internal stress).*

Supplementary Text: Mechanism of action

Here, we analyze the mechanism of SPOT more deeply, aiming to maximize the process's beneficial features. Specifically, SPOT results from the differential expansion between the different layers, which generates interlaminar stresses at the interface between the substrate and the nanomaterials/PVDF film.

Due to the high CTE value of PVDF (α = 127 × 10^{-6} K^{-1}), its layers experience extensive shrinkage during cooling. If the PVDF layer is attached to a substrate with a lower CTE, then we have a bimaterial membrane that deforms out of plane (bending) during the cooling phase. The out-of-plane motion is responsible for the generation of the out-of-plane stresses at the interface between the two materials, which in turn induces delamination and subsequent self-

peel-off behavior. The curvature of the bimaterial membrane (and as a consequence, the intensity of the residual stresses) depends on both the material's parameters and geometrical parameters, such as the thickness ratio between the different layers and the contrast in the elastic modulus between the layers. Timoshenko proposed an analytical solution based on thin structures bimaterial(Timoshenko, 1953) [S1]:

$$\frac{1}{R_T} - \frac{1}{R_{T_0}} = \frac{6(\alpha_2 - \alpha_2)(1+m)^2}{3(1+m)^2 + (1+m\bullet n)(m^2 + \frac{1}{m\bullet n})} \bullet \frac{T - T_0}{s} \qquad \text{(S1)}$$

where R_T is the radius of curvature at temperature T and R_{T0} is the radius of curvature at the other temperature, T_0, material 1 has the lower CTE (α_1) and material 2 has the higher CTE (α_2). $m = \frac{t_1}{t_2}$ is the thickness ratio between the two layers and $n = \frac{E_1}{E_2}$ is the the ratio of the elastic moduli of the materials. s is the total thickness of the film ($t_1 + t_2$).

It is clear from equation S1 that the change in radius of curvature is directly proportional to the change in temperature and the difference in CTE values. Moreover, a narrower thickness also increases the bending of the film. In other words, to maximize bending in a given temperature change, the bimaterial film must in theory be as thin as possible.

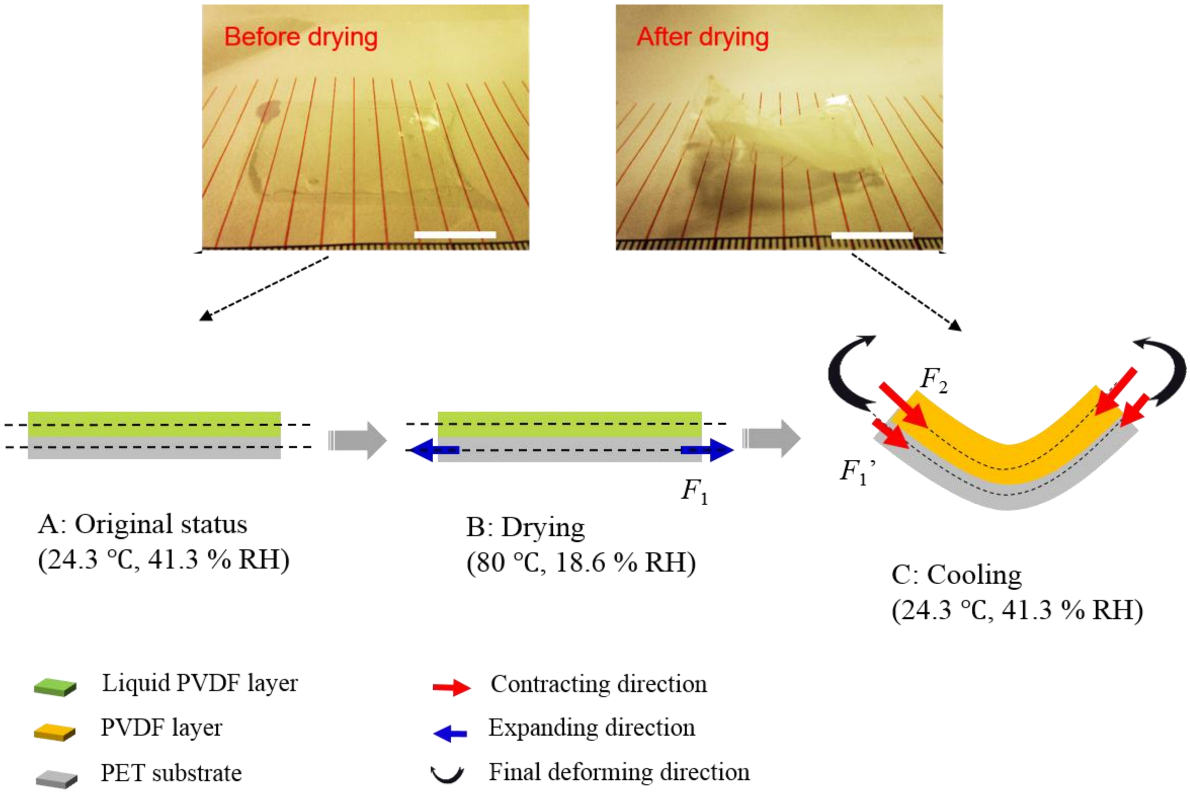

Figure S1. Digital images and schematics of the typical variation in the interfacial force and film deformation before and after drying. All the scale bars are 1 cm.

Table S1. The basic physical parameters of the materials used in this study.

**Material**	**Young's Modulus GPa 20 ℃ - 100 ℃**	**Poisson's ratio**	**Surface Energy Dynes/cm**	**CTE 10^{-6} K^{-1}**	**CHE % RH^{-1}**
Polyvinylidene fluoride	8.3	0.18	25	127	2.2×10^{-13}
Polyethylene terephthalate	3-5	0.3-0.4	41-44	59.4	$5\text{-}8 \times 10^{-6}$
Polydimethylsiloxane	0.0007/0.0009	0.5	22-24	310	NA
Glass	70	0.2	47	9	0
Graphene [S2, S3]	1000	0.33	8.64 ± 1.1	-8	NA
Graphene oxide [S3, S4]	208	0.197	57.1 ± 3.3	-67	NA
Carbon nanotube [S5, S6]	1000	0.1-0.55	10.2	-1.5	NA
Gold	69	0.42	NA	14.2	0
Palladium	121	0.39	NA	11.8	0
Poly(3,4ethylenedioxythiophene) polystyrene sulfonate	2.3	0.35	46.64	NA	0

Table S1 provides important physical parameters of the materials used in this study to confirm differences among different layers of polyvinylidene-fluoride (PVDF), nanomaterials and substrates.

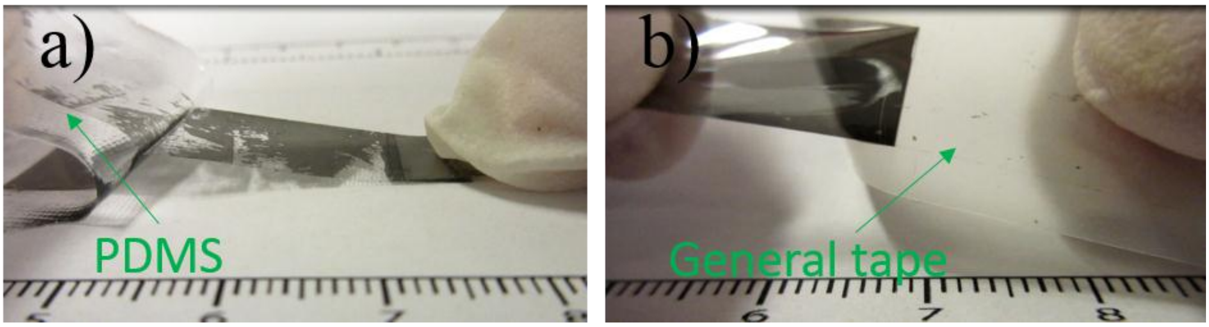

Figure S2 Other polymer materials used to transfer the Au-Pd film via self-peel-off transfer. A) PDMS; b) typical adhesive tape. The Au-Pd layer was deposited on PET substrate via sputter with a current of 20 mA for 180 s; PDMS liquid (curing agent/base = 1:10) was dropped on the Au-Pd layer with a controlled thickness of 300 μm after curing at 100 ℃ for 1 hour. This test verifies the advantages of PVDF for SPOT.

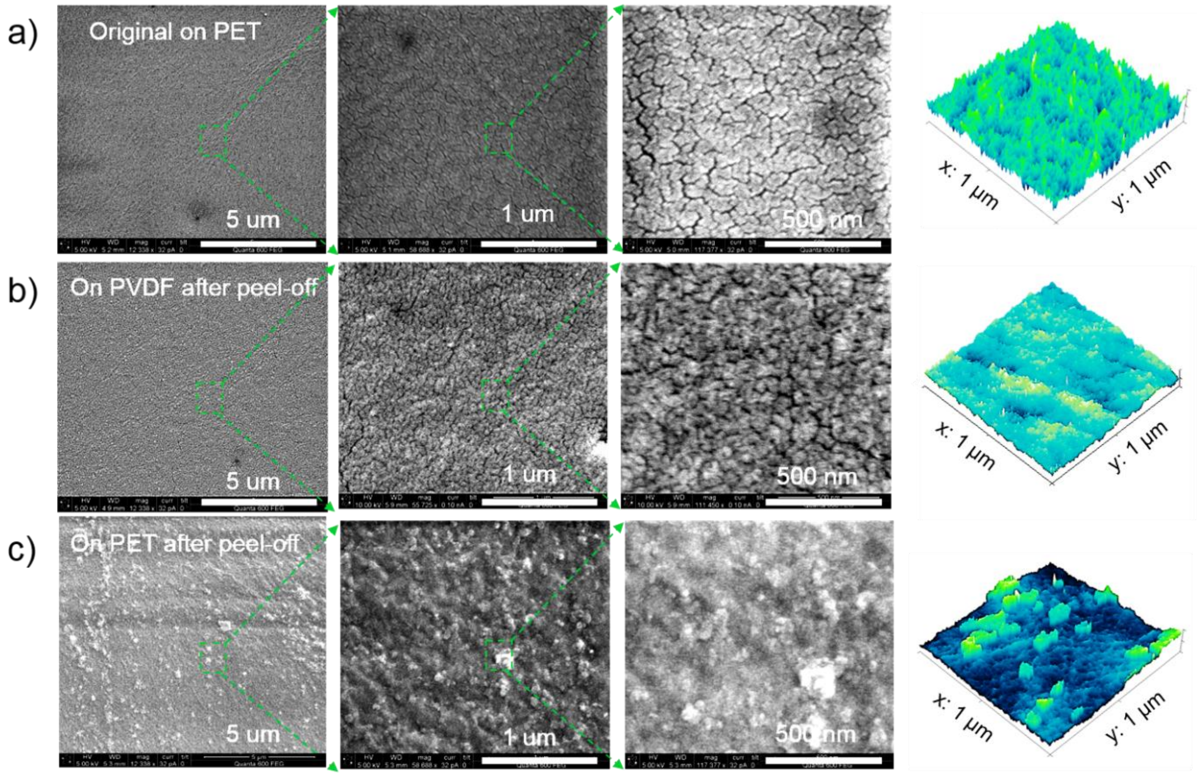

Figure S3. Morphology of the Au-Pd film before and after peel-off on different substrates (SEM images and AFM images), providing additional confirmation of the efficiency of SPOT: a) original Au-Pd film on PET; b) Au-Pd film on PVDF after peel-off; c) Au-Pd film on PET after peel-off.

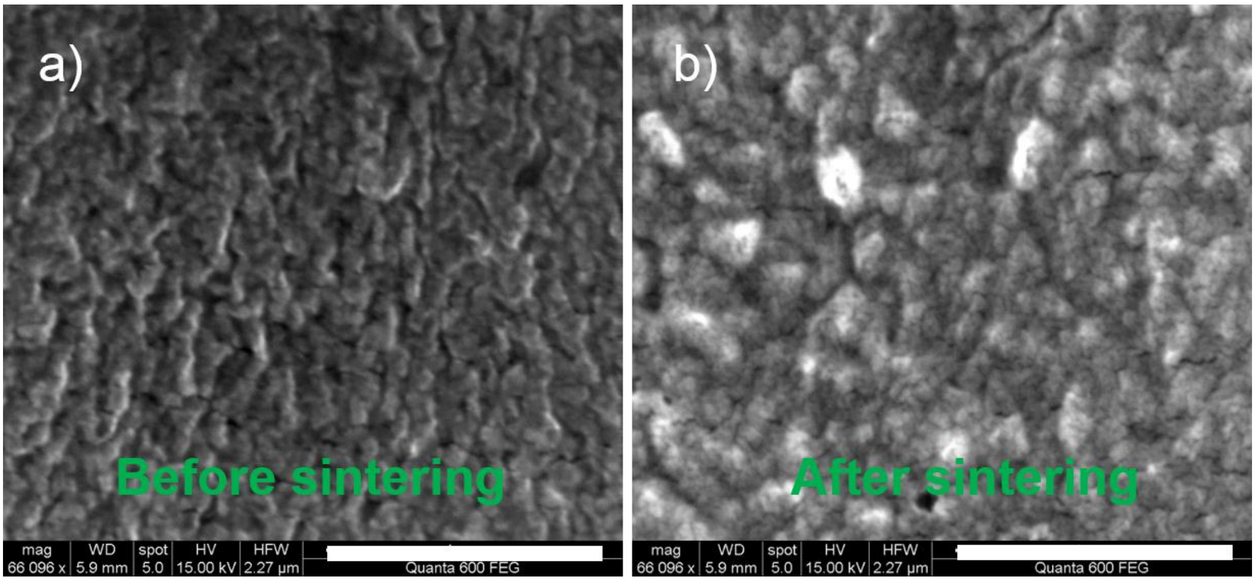

Figure S4. The microstructures of the Au-Pd layer on PVDF substrate. a) before and b) after sintering at 150 ℃ for 1 hour. The scale bars are1 µm.

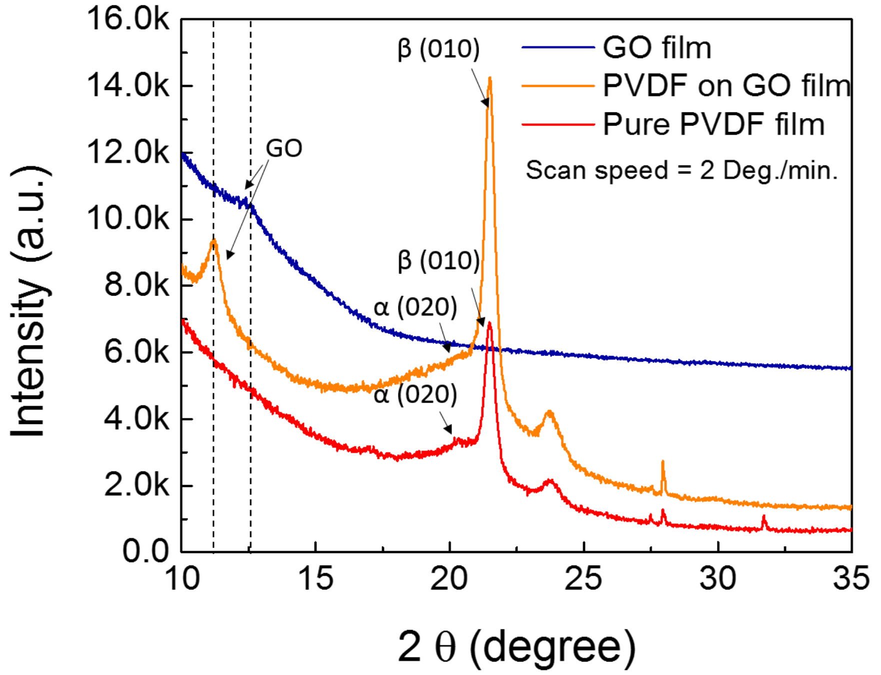

Figure S5. XRD pattern of a pure PVDF film, aPVDF film on GO film, and a GO film.

X-ray Diffraction (XRD) was also used to further verify the above prediction, as shown below. It can be found that, compared with the XRD pattern of pure PVDF, the PVDF film deposited on graphene oxide (GO) has a weaker alpha peak (α) and a stronger beta peak, indicating the relative reaction between the chemical groups on GO and the fluorine group in the PVDF chain, which creates a positive affect on the polarization of PVDF during its drying process. Meanwhile, the behavior can be further confirmed through the shift of a typical GO peak from 12.6 deg to 11.3 deg.

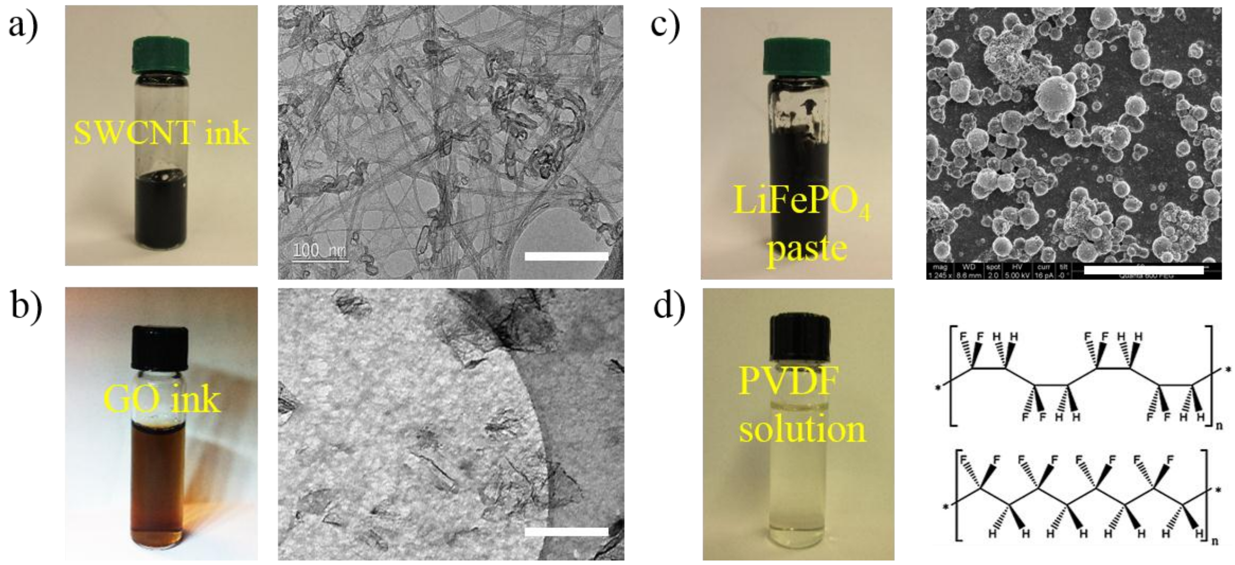

Figure S6. The main functional materials used in the manuscript. a) SWCNT ink (1 mg/ml) with TEM image; scale bar is 100 nm; b) GO ink (1 mg/ml) with TEM image; scale bar = 20 nm; c) $LiFePO_4$ paste with SEM image; scale bar = 50 µm; d) PVDF solution in DMF with its two typical chemical structures. [S7]

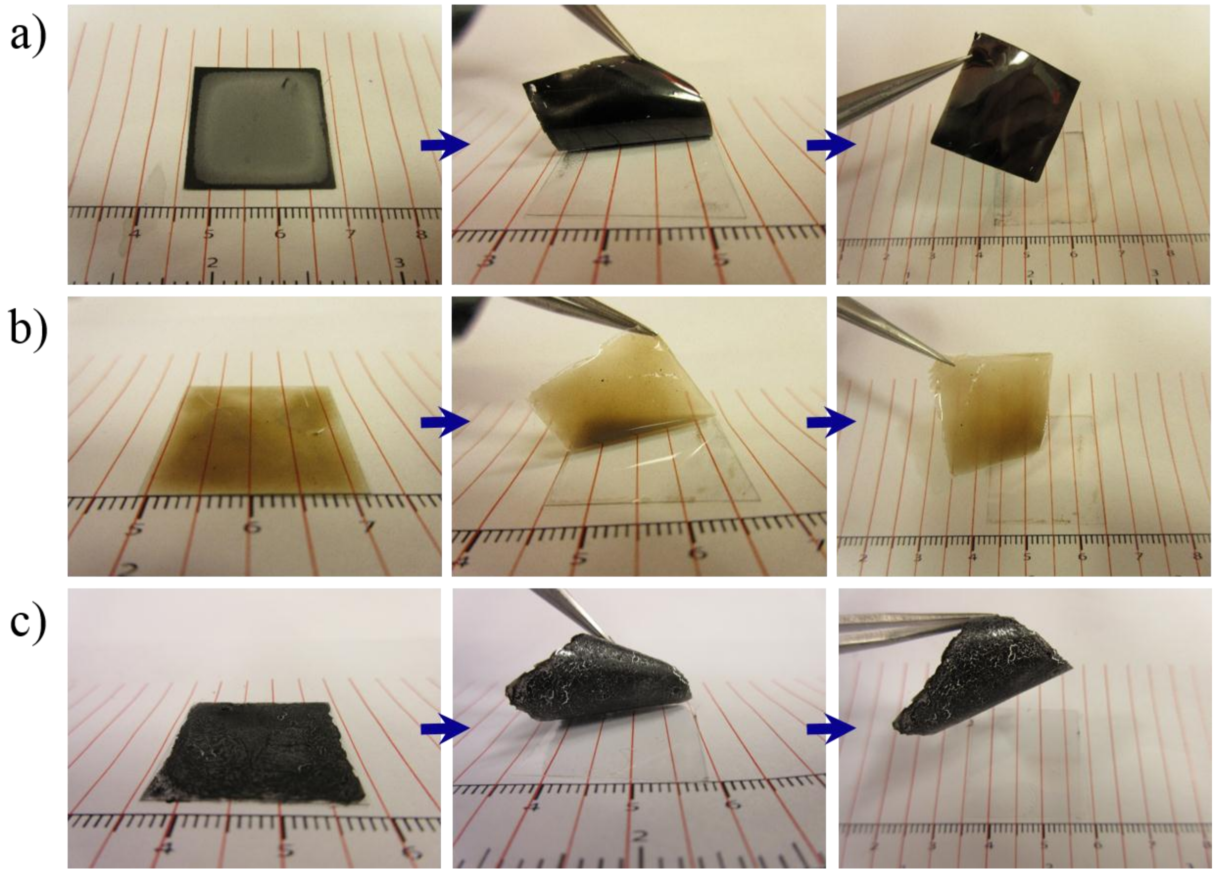

Figure S7. Preparation of various flexible PVDF functional films via SPOT. a) SWCNT; b) GO; c) $LiFePO_4$. Note that before the functional ink is dropped on the square glass slide (2 cm × 2 cm), the slide is cleaned with ethanol and treated with plasma.

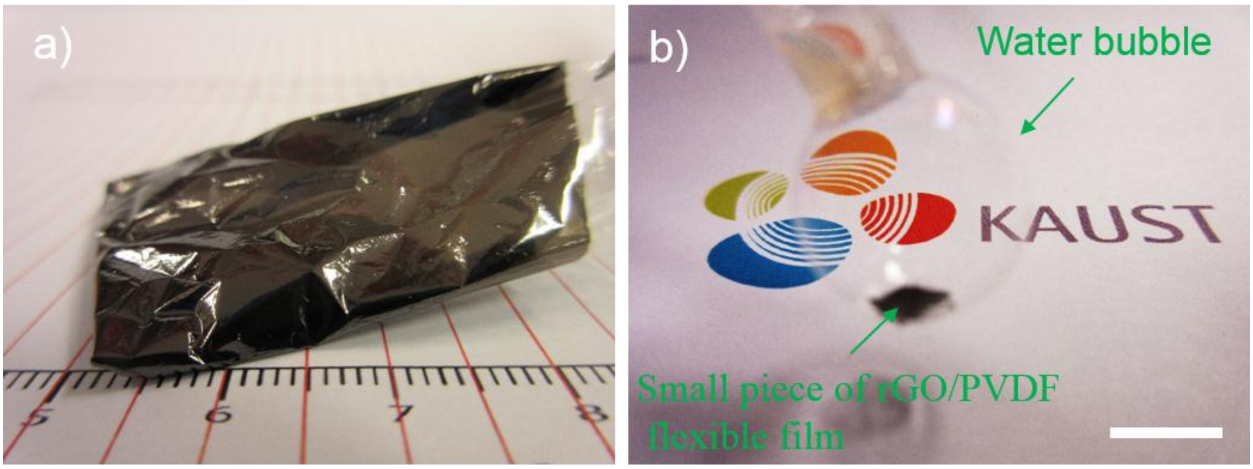

Figure S8. a) A typical large-area PVDF rGO film prepared via SPOT with a total thickness of 3 ± 0.5 µm; b) a demonstration of a small piece of a flexible rGO/PVDF film attached to a water bubble, indicating its ultra-light performance.

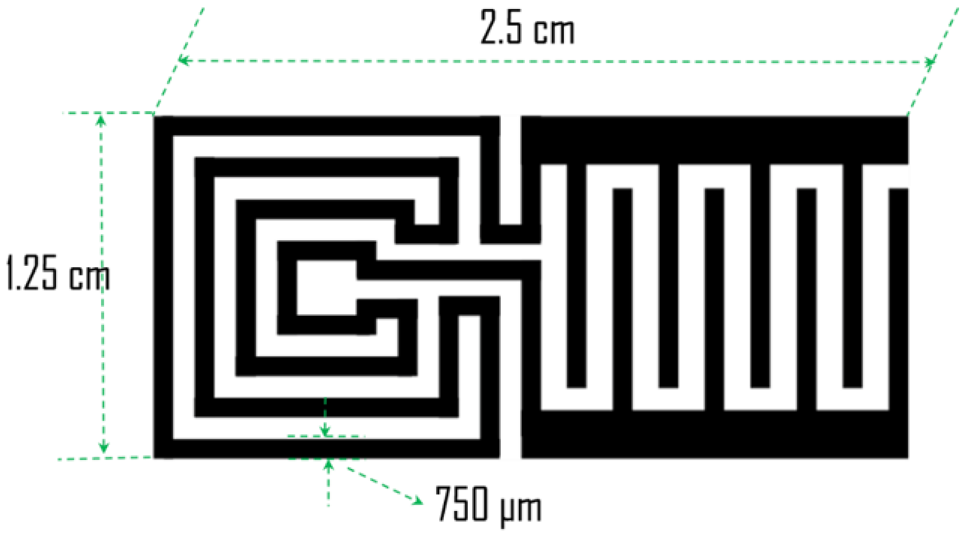

Figure S9. A schematic of a typical printed antenna with a total length of 2.5 cm and total width of 1.25 cm (the width of the conductive line = 750 µm and interval = 750 µm).[S8]

References

[S1] Timoshenko, S. History of strength of materials: with a brief account of the history of theory of elasticity and theory of structures. Courier Corporation. 1953.

[S2] Y. Duhee, et al. Negative thermal expansion coefficient of graphene measured by Raman spectroscopy. Nano letters 2011, 11, 3227.

[S3] J. Dai, et al. Study on the surface energies and dispersibility of graphene oxide and its derivatives. Journal of Materials Science 2015, 50, 3895.

[S4] Y. Kwon, et al. Thermal contraction of carbon fullerenes and nanotubes. Physical review letters 2004, 92, 015901.

[S5] D. A. Dikin, et al. Preparation and characterization of graphene oxide paper. Nature 2007, 448, 457.

[S6] S. Nuriel, et al. Direct measurement of multiwall nanotube surface tension. Chemical Physics Letters 2005, 404, 263.

[S7] Y. L. Tai, et al. Flexible, Transparent, Thickness-Controllable SWCNT/PEDOT: PSS Hybrid Films Based on Coffee-Ring Lithography for Functional Noncontact Sensing Device. Langmuir 2015, 31, 13257.

[S8] M. S. Mannoor, et al. Graphene-based wireless bacteria detection on tooth enamel. Nature communications, 2012, 3, 763.
